# Epigenomic Remodeling in Huntington’s Disease—Master or Servant?

**DOI:** 10.3390/epigenomes4030015

**Published:** 2020-07-31

**Authors:** Geraldine Zimmer-Bensch

**Affiliations:** Division of Functional Epigenetics in the Animal Model, Institute for Biology II, RWTH Aachen University, Worringerweg 3, 52074 Aachen, Germany; zimmer@bio2.rwth-aachen.de

**Keywords:** DNMT1, PCR2, histone modifications, histone variants, DNA methylation, transcriptional dysregulation, HTT, REST, MeCP2

## Abstract

In light of our aging population, neurodegenerative disorders are becoming a tremendous challenge, that modern societies have to face. They represent incurable, progressive conditions with diverse and complex pathological features, followed by catastrophic occurrences of massive neuronal loss at the later stages of the diseases. Some of these disorders, like Huntington’s disease (HD), rely on defined genetic factors. HD, as an incurable, fatal hereditary neurodegenerative disorder characterized by its mid-life onset, is caused by the expansion of CAG trinucleotide repeats coding for glutamine (Q) in exon 1 of the huntingtin gene. Apart from the genetic defect, environmental factors are thought to influence the risk, onset and progression of HD. As epigenetic mechanisms are known to readily respond to environmental stimuli, they are proposed to play a key role in HD pathogenesis. Indeed, dynamic epigenomic remodeling is observed in HD patients and in brains of HD animal models. Epigenetic signatures, such as DNA methylation, histone variants and modifications, are known to influence gene expression and to orchestrate various aspects of neuronal physiology. Hence, deciphering their implication in HD pathogenesis might open up new paths for novel therapeutic concepts, which are discussed in this review.

## 1. Introduction

Neurodegenerative diseases refer to a complex range of disorders characterized by neuronal disabilities and degeneration in the central or peripheral nervous system [1]. The innumerable potential mechanisms of neurodegeneration involve, amongst others, defects in protein homeostasis, impaired protein degradation, alterations in gene expression and transcriptional regulation, as well as mitochondrial dysfunction [2]. In order to improve early diagnosis and development of disease-modifying therapy strategies, we need to deepen our understanding of the interplay of pathophysiological mechanisms.

Epigenetic mechanisms call increasing attention in the field of neurodegeneration, having been investigated in the initiation and progression of neurodegenerative disorders, and even proposed as potential targets for therapeutic interventions [3]. Epigenetic regulation of gene expression is known to orchestrate diverse aspects of brain development including cell fate and neuronal differentiation, as well as survival regulation of post-mitotic and differentiating neurons. Epigenomic signatures are likewise dynamically reconfigured in the adult brain, being implicated in memory acquisition and consolidation by the modulation of synapse function through diverse mechanisms [4]. Epigenetic mechanisms are further suggested to act on age-related alterations in the brain like the loss of synapses and distinct neuronal populations [5,6]. For this, it is not surprising that epigenetic dysregulation has been proposed to be implicated in events underlying neuronal death in neurodegenerative diseases.

Indeed, over the past decade rising interest in the roles of epigenetic factors in pathological mechanisms of neurodegenerative diseases became evident [7,8]. This is likewise true for Huntington’s disease (HD), as the mutated huntingtin protein (HTT) has been reported to affect a wide range of epigenetic signatures, including histone modifications (i.e., acetylation, methylation and ubiquitination), and DNA methylation marks [8,9]. These changes can be related to transcriptional dysregulation, representing another hallmark of HD that is proposed to contribute to ensuing disease phenotypes. Albeit HD was so far thought to represent a single gene disorder, enormous variability in matters of disease onset and severity, even in patients with identical CAG trinucleotide repeat numbers, has been reported. Hence, in addition to the genetic cause, environmental factors are hypothesized to influence the susceptibility, onset, and progression of HD. These can be integrated by epigenetic mechanisms, known to be responsive to environmental stimuli.

The different epigenomic modifications cannot be regarded as separate issues. There is a high degree of crosstalk and interactions between the different chromatin modifying mechanisms [10,11]. While certain histone modifications prevent or promote DNA methylation, writers of DNA methylation can in turn be part of histone modifying complexes or modulate histone modifications via DNA methylation-dependent transcriptional control. Non-coding RNAs likewise exert transcriptional control in part by recruiting or preventing the binding of proteins/complexes involved in setting up DNA methylation and histone modification marks [12]. Hence, to explore the potential that epigenetic mechanisms have as targets for therapeutic interventions, we need to draw a conclusive picture, shedding light on the hierarchy of mutant HTT-induced epigenomic remodeling, and the precise contribution to the transcriptional dysregulation seen in HD.

## 2. Pathophysiological Features of Huntington’s Disease (HD)

Clinical symptoms of HD are progressive motor and cognitive decline, and psychiatric disturbances with the age of onset between 30 and 50 years, being fatal after 15–20 years of progressive neurodegeneration [13].

The predominant cause for HD—an autosomal dominant genetic disease—is a trinucleotide expansion (CAG) mutation in the 5′-coding region of the gene that encodes Huntingtin (HTT), resulting in polyglutamine (polyQ) repeats in the protein. After being investigated for two decades, both the exact function of unmutated HTT in healthy cells as well as the pathomechanisms of mutant HTT are still not sufficiently clarified. What is known is that these polyQ repeats cause misfolding of the mutant HTT protein, which is highly prone to aggregate and to form intracellular inclusion bodies [14,15,16,17,18]. In addition to the presence of mutant HTT aggregates, the gross atrophy of neostriatal nuclei, the caudate nucleus, and putamen accompanied by marked neuronal loss and astrogliosis, represent neuropathological characteristics of this disease [8]. Thereby, neuronal subtypes are selectively affected, with medium-sized spiny neurons (MSN) being most vulnerable at the earliest stage of disease [8]. MSNs represent gamma-aminobutyric acid (GABA)-positive striatal projection neurons morphologically characterized by a long axon, medium-sized cell bodies, and spiny dendrites [19,20,21]. Striatal interneurons that display unusually short axons, are typically not or only mildly affected at late stages of the disease. Although less pronounced than in the striatum, loss of particular neuronal populations is further observed in the cerebral cortex of HD patients, affecting large pyramidal projection neurons of cortical layers V and VI, and, less severe, layer III projection neurons [22,23]. Similar to the striatum, a noteworthy preservation of small cortical interneurons (i.e., layer IV granule cells) was observed in HD [22,24].

Remarkably, among different HD models behavioral and motor abnormalities were seen well before apparent neuronal degeneration [25,26,27], and prior to formation of polyQ-HTT aggregates [27]. This is in line with functional MRI imaging studies, that revealed early changes in neuronal function in pre-symptomatic HD patients [28], as well as with studies proposing a neuronal cell loss-independent early manifestation of chorea [29]. This indicates that the symptoms might not be primarily caused by the prominent neuronal loss and aggregate formation, but rather by preceding changes in neuronal physiology such as critical pathogenic events. In support of these observations, a marked reduction in the number of axonal fibers and synaptic proteins early in the course of HD was reported [25,30,31,32]. In line with that, early signs of axonal degeneration in the white matter of pre-symptomatic HD patients were revealed by diffusion tensor imaging studies [33,34]. The reported structural changes are consistent with progressive electrophysiological disturbances observed in association with polyQ-HTT expression [35,36,37,38]. Hence, alterations in neuronal physiology and connectivity seem to precede cell death in HD, with neurons following a “dying back” pattern of degeneration [31,39,40,41].

Similar to the delirious and toxic effects of the mutant HTT protein, the deletion of the normal *Htt* gene is likewise fatal in mice, implicating that proper HTT function is crucial for neuronal survival [8]. It is known so far that HTT represents a cytoplasmic protein ubiquitously expressed in neurons throughout the brain. Thereby, it is still a matter of debate how a ubiquitously expressed protein leads to selective neurodegeneration of particular neuronal subtypes. HTT was hypothesized to be implicated in intracellular transport, autophagy, transcription, mitochondrial function, and signal transduction, based on studies addressing the effects of both mutant HTT as well as wild-type HTT [8,42]. Thereby, its function seems not restricted to mature neurons as lowering wild-type *Htt* expression in the developing mouse brain leads to defects in cortical neuron migration [43], abnormalities in cortical and striatal synaptic development [43], and reduced cortical and striatal size and neuron numbers [44]. The cortical neurons show reduced brain-derived neurotrophic factor (*Bdnf*) expression and reduced activation of BDNF signaling pathways [44], which are consistent with the finding of diminished *BDNF* mRNA and protein levels in HD patients [43]. This indicates that wild-type HTT is relevant for neuronal development, which was recently proven by a study analyzing tissue from human fetuses that carry the Huntington’s disease mutation. The developing cortex displayed clear abnormalities. Apart from mis-localized mutant huntingtin and junctional complex proteins, defects in cell polarity and differentiation of neuronal progenitors, changes in mitosis and cell cycle progression, as well as abnormal ciliogenesis was seen. Similar observations were made in Huntington’s disease mouse embryos, where the abnormalities were linked to defects in interkinetic nuclear migration of progenitor cells [45]. Hence, clearly dissecting the function HTT has in neurodevelopment from its function in adult neurons appears essential, especially as there is an increasing line of evidence that neurodegeneration is linked to or primed by neurodevelopmental processes.

In addition to its cytoplasmic localization, HTT is evident in the nucleus, capable of interacting with DNA, transcription factors and multi-subunit complexes [46,47,48,49,50]. Thus, altered interactions of mutant HTT with proteins of the transcriptional and epigenetic machinery could lead to the transcriptional dysregulation frequently reported for HD, and through this affect a diversity of processes underlying proper neuronal physiology.

## 3. The Epigenetic Toolkit

The epigenetic toolkit encompasses histone variants and modifications, DNA methylation, alterations in nucleosome positioning and non-coding RNAs.

The DNA is wrapped around histone octamers, composed of the highly conserved H2A, H2B, H3, and H4 histone proteins, forming the repeated basic unit of chromatin—the nucleosome. Apart from replication dependent canonical histones, distinct histone variants exist. These variants can either display minor sequence differences (e.g., the canonical H3.1 and H3.2, and the variant H3.3) or significant structural dissimilarities (e.g., macroH2A, the centromere-specific protein CENP-A). Histone variants exhibit different cell-type specific expression levels [51], and undergo posttranslational histone modifications like the canonical histones [52,53]. Incorporation of certain histone variants can affect gene transcription, whereas the effect on transcription depends on the incorporated variant, the site of insertion in the genome as well as the variant-specific posttranslational modifications.

Histone modifications occur mostly at the N-terminal tails of the core histones. These posttranslational modifications include acetylation, methylation, phosphorylation, SUMOylation and ADP-ribosylation [2], which are dynamic and reversible processes. Histone modifications are achieved by two antagonistic sets of enzyme complexes that either attach (writers) or remove (erasers) the respective chemical groups. Acetylation at lysine residues, as one of the most intensively studied histone modification, is associated with transcriptional activation [54]. Histone acetyl transferases (HATs) add acetyl groups, whereas histone deacetylases (HDACs) remove them. Histone methylation can either be associated with transcriptional repression or activation, dependent on the site, which is subject to methylation, as well as the degree of methylation. While H3K4-trimethylation (me3) leads to open chromatin, H3K27me3 causes chromatin condensation associated with gene repression [55].

DNA methylation is catalyzed by DNA methyltransferases (DNMTs), of which DNMT1 and DNMT3A are mainly expressed in the adult brain. DNA methylation can occur at enhancer and promoter sites, as well as in gene bodies and intergenic regions, being associated with transcriptional regulation [56,57]. Of note, gene body methylation was recently reported to be involved in alternative splicing and alternative promoter choice [58,59]. The mechanisms, through which DNA methylation acts on gene transcription, emerge to be very diverse. Transcription factor binding can be physically impeded when the DNA is methylated. Moreover, methyl-CpG-binding domain proteins (MBDs) interact with methylated DNA, which can recruit other chromatin and nucleosome remodeling proteins, that concertedly lead to compact and inactive heterochromatin [60]. This is consistent with the textbook model of repressive DNA methylation in gene promoter regions. In contrast to the traditional view of DNA methylation preventing the binding of proteins without a methyl-CpG-binding domain (MBD), numerous reports indicate that DNA methylation signatures can even serve as binding motifs for particular transcription factors that lack a methyl binding domain [61]. Based on in silico studies, an increasing pool of transcription factors is suggested to bind methylated DNA sequences. Newly emerging scenarios propose that DNA methylation signatures could create new binding motifs for certain transcription factors. Moreover, transcription factors might even recognize different sequences dependent on whether they are methylated or not [62].

Alike histone modifications, DNA methylation is a dynamic process. In addition to passive DNA demethylation in dividing progenitors, ten-eleven translocation (TET) family enzyme-dependent mechanisms enable active ways of DNA demethylation in non-dividing cells [63,64]. Through TET-mediated oxidation of 5-methylcytosine (5mc) to 5-hydroxymethylcytosine (5hmc) and iterative oxidation forms, active reversion to cytosine can be achieved by thymine DNA glycosylase (TDG)-mediated base excision repair [64,65,66], which is also seen in neurons [67,68].

Non-coding RNAs are distinguished in small and long non-coding RNAs (scnRNAs and lncRNAs, respectively), differing in size, biogenesis, and function. The function of sncRNAs, including miRNAs, siRNAs and piRNAs, mainly refers to posttranscriptional regulation in the cytoplasm [12]. In contrast, the functional spectrum of lncRNAs emerged to be tremendously diverse, modulating transcriptional, post-transcriptional and even translational events [12].

Through intensive crosstalk, the different mechanisms concertedly modulate chromatin structure and gene expression. Certain histone modifications can, for example, predispose for the establishment of DNA methylation marks and vice versa [11,69,70]. Furthermore, DNMT1 was shown to influence histone modifications by regulating the transcription of genes encoding for enzymes of histone modifying complexes as well as by interacting, e.g., with the PRC2 at protein level [71,72]. Moreover, lncRNAs were described to recruit or evict the binding of DNMTs and histone modifying complexes [12,73,74], thereby mediating target specificity. Hence, it should be kept in mind that a complex interplay between different epigenomic remodelers exists and concertedly orchestrates transcriptional regulation, thereby coordinating diverse physiological processes in neurons.

## 4. Implications of Epigenetic Mechanisms in the Regulation of Neuronal Survival and Functionality in the Healthy Brain

To unveil potential mechanisms, through which epigenetic mechanisms mediate disease-related neurodegeneration, their implication in the regulation of neuronal functionality and survival in healthy brains should be highlighted.

Neuronal survival regulation plays a pivotal role in the developing central nervous system, in which DNA methylation and DNMTs have been widely described to be involved [75,76]. Conditional deletion of *Dnmt1* in *Emx1*-expressing telencephalic precursors from embryonic day 13.5 on is accompanied by prominent hypomethylation and causes progressive cell death at post-mitotic level, leading to a subsequent reduction in cortical thickness [77]. This emphasizes a crucial role of DNMT1-dependent DNA methylation in promoting the survival of excitatory neurons during brain development. In line with that, conditional deletion of *Dnmt1* in post-mitotic migrating inhibitory interneurons causes elevated cell death rates, suggesting that DNMT1 likewise promotes the survival of the inhibitory subpopulation of the developing telencephalon [78]. *Pak6* and *Lhx1* were both identified as target genes, through which DNMT1 acts on interneuron survival [71,79,80]. However, the DNA methylating activity of DNMT1 emerged of subordinate relevance for *Lhx1* and *Pak6* dependent transcriptional control in the context of survival regulation, which seems to be rather achieved by a crosstalk of DNMT1 with histone methylation and acetylation [71,79]. In addition to DNA methylation and the interference of DNMTs with posttranslational histone modifications, lncRNA function is crucially implicated in neuronal survival regulation during brain development [81].

Similar to developing neurons, epigenetic mechanisms influence neuronal survival in the adult brain through a variety of mechanisms. Survival of both maturing as well as adult neurons depends on stimulation. Electrical activity promotes the calcium-dependent neuronal survival during development, which contributes to the removal of neurons that have not made appropriate synaptic connections (for review see [82]). Moreover, neuronal activity was also shown to modulate the survival of mature neurons [83]. Neuronal activity has been shown to change the DNA methylation landscape in the adult brain [84], and to trigger the expression of histone 3.3 variants, which is in turn involved in activity-dependent gene expression [85]. Besides, neuronal activity induces histone acetylation, that in turn shapes the transcriptional dynamics of neuronal activity-driven gene expression [86]. Hence, activity-driven remodeling of epigenomic marks might act on survival related gene expression.

The olfactory system provides an excellent example for activity-triggered survival regulation mediated by epigenetic mechanisms. As revealed by loss- and gain-of-function experiments, the histone variant H2be was reported to promote apoptosis of under-stimulated olfactory neurons, hence reducing their life span. This variant is only expressed in mature olfactory and vomeronasal neurons, differing by only five amino acids from the H2b isoform. The H2be variant, which is particularly enriched at euchromatic parts of the genome, can neither be acetylated nor methylated on lysine 5; both of these posttranslational histone modifications are related to transcriptional elongation [83]. Frequent activation of olfactory neurons in turn reduces H2be levels, which are associated with an extended life span [83]. However, the detailed molecular mechanism through which H2be modulates longevity is not yet understood. It is proposed that such a dynamic epigenetic regulation would provide each individual with a spectrum of olfactory neurons customized to their specific odorant environment, even though the initially expressed olfactory receptors were chosen stochastically [83,87].

Apart from under-stimulation, it is known that hyperexcitability likewise can lead to neuronal death [88], requiring a well-balanced regulation of neuronal activity. Neuronal activity relies on synapse function regulation, for which the implication of epigenetic mechanisms was already reported. In excitatory neurons of the cerebral cortex, DNMTs modulate synaptic transmission [4,89,90], synaptic scaling [91] and neuronal excitability [92]. Similarly, TET enzymes, initiating DNA demethylation, are implicated in synapse function regulation. TET1 dependent transcriptional control of activity regulated genes was shown to be involved in synaptic plasticity and memory extinction [93]. Moreover, TET3 acts on homeostatic plasticity by regulating gene expression in response to global synaptic activity alterations [94]. Recently, we have shown that DNMT1 dependent DNA methylation modulates synaptic transmission in inhibitory cortical interneurons by regulating synaptic vesicle recycling through the transcriptional control of endocytosis-related genes [95]. As synapse function and neuronal excitability regulation both influence neuronal activity [96] and hence, the vitality of neurons, epigenetic mechanisms could indirectly act on neuronal survival by modulating pre- and postsynaptic processes.

DNMT1 function was further reported to influence the survival of inhibitory interneurons in the aged cerebral cortex [5]. Deletion of *Dnmt1* in adult *Pvalb*-expressing cortical interneurons significantly prevents their age-related loss. This is accompanied by diminished age-associated transcriptional changes, as well as by improved motoric performances of aged conditional *Dnmt1* knockout mice [5]. However, a dysregulation of survival or cell-death related genes induced by *Dnmt1* deletion could not be identified as causative in the old interneurons. In turn, various genes related to the proteostasis network were affected by the loss of DNMT1 function in young mice. Functional validation studies revealed that DNMT1 negatively influences subcellular processes implicated in endolysosomal protein degradation pathways [5], as well as in autophagy [97]. In line with that, defects in endocytosis, and endolysosomal protein degradation have already been described to be involved in age- and disease-related neurodegeneration [98,99,100,101]. Thus, DNMT1-dependent transcriptional control could act on neuronal long-term survival by impeding diverse facets of protein degradation over life-time. This mechanism could likewise be of relevance for neurodegenerative diseases, in which defects in the protein degradation machinery as well as epigenomic signatures were frequently reported to be of importance.

## 5. Epigenetic Mechanisms in HD

Transcriptional dysregulation has often been described to be implicated in neurodegenerative diseases, which may be, at least in part, caused or mediated by epigenetic changes. Transcriptional dysregulation, being evident across diverse HD models [102], accounts as a major pathogenic mechanism of HD and an early event in HD pathology, preceding the onset of neuronal cell death [9,102]. HTT is thought to be a multi-functional protein, suggested to participate in diverse cytosolic processes, like vesicle trafficking, as well as being localized in the nucleus and binding to the DNA, thereby influencing transcription [46]. Moreover, HTT was described to associate with diverse transcription factors [50]. Hence, it is proposed that the polyglutamine (polyQ) repeat expansion affects the interaction of HTT with transcriptional regulators, whereby their normal function is disrupted and transcriptional dysregulation could be mediated.

One crucial transcription factor HTT interacts with is REST (Repressor Element 1-Silencing Transcription factor). REST is suggested to act as a hub orchestrating transcriptional and epigenetic programs, of which many are impaired in HD. Numerous non-coding RNAs, including both microRNAs and long non-coding RNAs, several of which are dysregulated in HD, represent key target genes of REST [48]. Moreover, REST also recruits a diversity of histone and chromatin modifying activities, that can regulate the local epigenetic signature at REST target genes [48]. Wild-type HTT sequesters REST in the cytoplasm of vulnerable cells within the striatum in mice. The trinucleotide-repeat expansion in mutant HTT disrupts this interaction, causing REST to translocate to the nucleus, triggering the expression of several epigenetic key players in HD, which likely contributes to the transcriptional dysregulation.

In addition to REST, full-length HTT is described to function as a facilitator of macromolecular complexes, such as the multi-subunit polycomb repressive complex 2 (PRC2) complex [49] in the nucleus, which acts as an epigenetic silencer by catalyzing repressive H3K27 trimethylations [103]. Full-length recombinant HTT promotes the histone H3K27 tri-methylase activity of PRC2 in vitro. This effect was progressively elevated with increasing numbers of polyglutamine-repeats [49]. Hence, PRC2 represents a direct target of HTT, and mutated HTT might lead to transcriptional dysregulation by altered modulation of PRC2 activity. The connection between HTT and PRC2 activity was further supported by expressing mutant *Htt* in embryonic stem cells, which causes a genome-wide changed pattern of H3K27me3 [47]. Induced PRC2-deficiency in striatal medium spiny neurons (MSNs) affects death-promoting proteins, in addition to PRC2 target genes, that encode transcription factors, which are normally suppressed in MSNs. These changes in gene expression are accompanied by progressive and fatal neurodegeneration [104], emphasizing the connection between mutant HTT, PRC2 and neurodegeneration. Besides, loss of PRC2 in forebrain neurons triggers increased expression of genes involved in HD [104].

In addition to histone methylation, mutant HTT was further described to alter histone acetylation [105] and ubiquitylation [106], which are linked to transcriptional control as well [107]. HTT was found to bind the histone acetyltransferase domain of the CREB-binding protein (CBP), leading to histone hypoacetylation [108] and gene repression [105]. In line with that, protein aggregates constituting plaques found in the brains of patients with HD and AD were reported to contain sequestered proteins with histone acetyltransferase activity (notably CBP), which potentially leads to diminished histone acetylation [109]. For this, HDAC inhibitors are discussed as a potential therapeutic strategy for HD (reviewed in [8]).

Apart from this crosstalk with transcription factors and histone modifying complexes, HTT protein was described to directly interact with MeCP2 [110]. MeCP2 belongs to the methyl-binding domain-containing (MBD) proteins, acting as reader and effector of DNA methylation. Through binding of methylated DNA, MeCP2 recruits transcriptional co-repressors to silence transcription by modifying the chromatin structure [111]. Recently, MeCP2 has been shown to additionally act as a transcriptional activator via interaction with CREB [112]. Hence, MeCP2 provides an essential link between DNA methylation, chromatin remodeling and dynamic gene expression [113,114]. Due to the expanded polyglutamine (polyQ) tract, the interactions of MeCP2 with mutant HTT are enhanced, and stronger apparent in the nucleus compared to the cytosol [110]. As described above, HTT interacts with chromatin modifiers and mutant HTT leads to altered epigenetic marks [8]. Similarly, MeCP2 represents a multifunctional regulator of gene expression. Hence, the increased HTT-MeCP2 interactions in the nucleus, occurring as a consequence of the expanded polyglutamine repeats, in turn may elicit a diverse spectrum of epigenomic and transcriptional changes. In the presence of mutant HTT, the binding of MeCP2 to the promoter of *BDNF*, a gene that is downregulated in HD, was increased [110]. Together, this suggests that the aberrant interactions between mutant HTT and MeCP2 are likely implicated in the transcriptional dysregulation seen in HD.

In addition to changed histone marks, altered DNA methylation signatures have been reported in HD patients and transgenic mice, including changes in 5-methylcytosine (5mC) and 5-hydroxymethylcytosine (5-hmC) patterns [115,116,117]. DNA methylation changes were identified at promoter, proximal and distal regulatory regions in mouse STHdhQ111 cells compared to WTQ7 cells, with some sites of increased, as well as decreased, methylation levels [115]. Changes in DNA methylation detected at CpG-rich regions, which were found mostly located close to transcription start sites, were revealed to be inversely correlated with gene expression [115]. The affected genes are related to developmental processes, such as neurogenesis, neuron migration, and cell differentiation. Identified neurogenesis associated genes, including *Ap-1, Sox2, Pax6*, and *Nes*, were found highly methylated, while their expression was significantly reduced. Based on this, it is hypothesized that a DNA methylation dependent impairment in hippocampal neurogenesis is mechanistically associated to the cognitive decline seen in HD patients [115]. However, this requires verification in HD patients.

Another gene which displays altered DNA methylation signatures in HD patients and HD transgenic mouse models is *ADORA2A*, encoding for the adenosine A2A receptor (A2AR). A2AR is a G-protein-coupled receptor, that is normally highly expressed in the basal ganglia, but found to be severely reduced in HD [116]. Diverse pharmacological therapies have already provided evidence for its implication in HD disease pathology. In HD patients, the diminished expression appears correlated with increased levels of 5-mC in the 5′-UTR region of *ADORA2A* gene in the striatum. In contrast, in HD transgenic mice, decreased 5-hmC levels in the 5′-UTR region of the *Adora2a* gene were described [116]. Albeit differentially modulated between species, the alterations in DNA methylation signatures in the *ADORA2A* gene are proposed as a pathological mechanism putatively relevant to HD. Of note, genome wide marked reduction of 5-hmC was found in the postnatal brain tissue of an HD mouse model, pointing to a deficiency of 5-hmC reconstruction in the brain as a crucial feature of HD [118].

Oxidation of 5-mC to 5-hmC is critically involved in active DNA demethylation, hence enabling dynamic DNA methylation-dependent transcriptional control [119]. Aberrant hydroxymethylation profiles and expression level were seen for genes that are implicated in neuronal development and differentiation (Wnt/β-catenin/Sox pathway, axonal guidance signaling pathway), as well as neuronal function and survival (e.g., glutamate receptor/calcium/CREB, GABA receptor signaling, dopamine-DARPP32 feedback pathway), in the striatum and cortex [118]. This altered epigenetic regulation links DNA methylation/demethylation-dependent transcriptional control to impaired neurogenesis and neuronal function, as well as to the neuronal death seen in HD. However, how these HD related alterations in DNA methylation and hydroxymethylation signatures are mechanistically realized, remains elusive so far.

One scenario could be that changes in DNA methylation pattern occur secondarily due to mutant HTT induced alterations in histone modifications. DNA methylation was found inversely correlated with H3K4me3 deposition and positively correlated with H3K9me3 as well as H3K27me3 marks, the latter being catalyzed by PRC2. H3K4me3 was suggested to prevent DNMT3-dependent DNA methylation [120]. H3K9me3 marks recruit DNMT3a/b by direct interactions with heterochromatin protein 1 (HP1), which binds to H3K9me3 through its chromodomain [121]. Hence, mutant HTT dependent changes in deposition and/or removal of histone marks either associated with heterochromatin or decondensed chromatin could lead to the recruitment of DNMTs or the prevention of their binding. Likewise, binding of key players like TETs or GAD65, involved in DNA demethylation, could be influenced secondarily. In support of this, loss of H3K4me3 at CpG-rich sequences on the HES4 was associated with excessive DNA methylation and altered expression of HES4, as well as of its target genes. Apart from that, hypermethylation of the HES4 promoter was associated to measures of striatal degeneration and age-of-onset in HD brains [122].

Changed expression of writers and erasers of DNA methylation was further suggested as a potential mechanism through which DNA methylation signatures are altered in HD. This is based on the observation that a downregulation of *Dnmt* genes was observed in some studies (reviewed in [123]). *Dnmt1* expression was found to be decreased two-fold in HD STHdhQ111 cells compared to WTQ7 cells [26], and diminished *Dnmt1* expression was observed in the striatum and cortex of N171-82Q transgenic HD mice compared to littermate controls. Reduced *Dnmt1* expression is assumed to correspond to lower levels of DNA methylation.

However, DNA methylation levels in HD models were not exclusively observed to be reduced. DNA methylation was also found elevated, e.g., increased *BDNF* promoter methylation was detected in blood cells of HD subjects [124], which is consistent with the reduced BDNF mRNA and protein levels detected in HD patients [125]. Similarly, in mutant HTT-expressing neurons, elevated levels of DNA methylation were found in the *Bdnf* promoter region, and inhibition of DNMTs in these primary cortical or striatal neuron HD models restored the expression of *Bdnf*, as well as of several other key genes implicated in HD pathophysiology [126]. So, how may the increase in DNA methylation be mediated in HD when *Dnmts* are rather found downregulated? It was proposed that diminished expression of genes encoding DNA demethylases could account for such elevation in the DNA methylation levels seen in HD subjects and model systems (summarized in Thomas, E.A (2016), [123]). The reduced expression of DNA demethylation-associated genes suggested to cause an increase in methylation, which could then lead to a downregulation of *Dnmts* as a secondary counter-regulatory effect. Decreasing *Dnmt* expression or impaired DNMT function seems favorable in the context of HD, as the DNMT inhibition as well as a knockdown of *Dnmt1* and *Dnm3a* was found to reduce mutant HTT-triggered neurotoxicity in primary cortical and striatal neurons [126]. Overall, this aspect again tackles the crucial issue of the hierarchy of mutant HTT-triggered epigenetic alterations, dissecting causes from consequences.

In general, it should be stated that global transcriptional changes in DNA methylation writers and erasers do not explain site-specific effects, as seen for genomic loci with increased versus decreased cytosine methylation. Yet, these findings concertedly demonstrate that DNA methylation is involved in mutant HTT-induced transcriptional dysregulation and neurotoxicity, whereas the exact mechanisms of how mutant HTT promotes DNA methylation at specific gene loci at the molecular level remains a significant, still open question. Apart from mutant HTT-triggered transcriptional regulation of the expression of writers or erasers of DNA methylation, it is conceivable that the modulation of their activity is affected by binding partners, similarly to what was described for PRC2 activity [49]. Besides, mutant HTT could facilitate the recruitment of the DNA methylation machinery to specific genomic regions, and/or prevent their binding at specific loci through the diverse repertoire of interactions with other epigenetic key players.

## 6. Conclusions

HD is a neurodegenerative disorder with known genetic cause, leading to extended polyglutamine repeats and hence, altered HTT functionality. In addition to this, changed epigenomic signatures were identified, which offers an attractive hypothesis to explain transcriptional dysregulation of the multiple genes seen in HD. As epigenetic mechanisms are responsive to environmental influence, they are proposed to contribute to the variability of disease onset and progression. Moreover, by cell type-specific actions, epigenetic mechanisms could further be implicated in mediating the cell type-specific vulnerability. The reversion of particular molecular and phenotypic features by treatment with chromatin-modifying drugs, having been shown to be beneficial at the preclinical stage [127,128,129], propose epigenetic key players to be promising targets for therapeutic intervention.

The studies performed in the HD context suggest that the changes in epigenetic modifications, rather than being the cause, apparently are a mere consequence of the underlying genetic mutation leading to altered HTT interactions with epigenetic key players like REST, PRC2 and MeCP2. This in turn results in a multitude of changes in epigenetic signatures, which—as stated above—can vary between different neuronal subtypes. As epigenomic reconfigurations influence numerous physiological processes, ranging from synaptic function to proteostasis regulation, the altered interactions of mutant HTT can elicit a variety of distinct cell type-specific physiological changes. The complexity of effects is even more increased by the diverse spectrum of crosstalk between the different epigenetic mechanisms. Hence, effective treatment and modifying disease pathology by therapeutically targeting specific epigenetic modifiers can only be achieved after deciphering the hierarchy of epigenetic changes, and the discrete subcellular processes which are affected in the different neuronal populations. For this, apart from investigating DNA methylation, histone modification and ncRNAs separately, integrative genome-wide analysis has to be performed cell type-specifically, to unveil the relation of the different epigenetic marks to each other, and to approach the underlying causes of the selective vulnerability of different neuronal subtypes. The enormous technological progress that is continuously achieved in the field of single cell sequencing, might bring this challenging goal in feasible reach.

## References

[1] Vila M., Przedborski S. (2003). Targeting programmed cell death in neurodegenerative diseases. Nat. Rev. Neurosci..

[2] Lovrečić L., Maver A., Zadel M., Peterlin B. (2013). The role of epigenetics in neurodegenerative diseases. Neurodegener. Dis..

[3] Xylaki M., Atzler B., Outeiro T.F. (2019). Epigenetics of the Synapse in Neurodegeneration. Curr. Neurol. Neurosci. Rep..

[4] Sweatt J.D. (2016). Dynamic DNA methylation controls glutamate receptor trafficking and synaptic scaling. J. Neurochem..

[5] Hahn A., Bayer C., Pensold D., Tittelmeier J., Marx-Bluemel L., Gonzalez-Bermudez L., Linde J., Gross J., Salinas-Riester G., von Maltzahn J. (2020). DNA methyltransferase 1 (DNMT1) function is implicated in the age-related loss of cortical interneurons. BioRxiv.

[6] Zimmer-Bensch G. (2019). Functional Implications of Dynamic DNA Methylation for the Developing, Aging and Diseased Brain. The DNA, RNA, and Histone Methylomes.

[7] Lardenoije R., Iatrou A., Kenis G., Kompotis K., Steinbusch H.W., Mastroeni D., Coleman P., Lemere C.A., Hof P.R., van den Hove D.L. (2015). The epigenetics of aging and neurodegeneration. Prog. Neurobiol..

[8] Lee J., Hwang Y.J., Kim K.Y., Kowall N.W., Ryu H. (2013). Epigenetic mechanisms of neurodegeneration in Huntington’s disease. Neurotherapeutics.

[9] Sugars K.L., Rubinsztein D.C. (2003). Transcriptional abnormalities in Huntington disease. Trends Genet.

[10] Backofen R., Vogel T. (2014). Biological and bioinformatical approaches to study crosstalk of long-non-coding RNAs and chromatin-modifying proteins. Cell Tissue Res..

[11] Du J., Johnson L.M., Jacobsen S.E., Patel D.J. (2015). DNA methylation pathways and their crosstalk with histone methylation. Nat. Rev. Mol. Cell Biol..

[12] Zimmer-Bensch G. (2019). Emerging Roles of Long Non-Coding RNAs as Drivers of Brain Evolution. Cells.

[13] Harper P. (1996). Major Problems in Neurology: Huntington’s Disease.

[14] DiFiglia M., Sapp E., Chase K.O., Davies S.W., Bates G.P., Vonsattel J., Aronin N. (1997). Aggregation of huntingtin in neuronal intranuclear inclusions and dystrophic neurites in brain. Science.

[15] Gutekunst C.-A., Li S.-H., Yi H., Mulroy J.S., Kuemmerle S., Jones R., Rye D., Ferrante R.J., Hersch S.M., Li X.-J. (1999). Nuclear and neuropil aggregates in Huntington’s disease: Relationship to neuropathology. J. Neurosci..

[16] Lunkes A., Lindenberg K.S., Ben-Haïem L., Weber C., Devys D., Landwehrmeyer G.B., Mandel J.-L., Trottier Y. (2002). Proteases acting on mutant huntingtin generate cleaved products that differentially build up cytoplasmic and nuclear inclusions. Mol. Cell.

[17] Schilling G., Klevytska A., Tebbenkamp A.T., Juenemann K., Cooper J., Gonzales V., Slunt H., Poirer M., Ross C.A., Borchelt D.R. (2007). Characterization of huntingtin pathologic fragments in human Huntington disease, transgenic mice, and cell models. J. Neuropathol. Exp. Neurol..

[18] Juenemann K., Weisse C., Reichmann D., Kaether C., Calkhoven C.F., Schilling G. (2011). Modulation of mutant huntingtin N-terminal cleavage and its effect on aggregation and cell death. Neurotox. Res..

[19] Gerfen C.R. (1988). Synaptic organization of the striatum. J. Electron Microsc. Tech..

[20] DiFiglia M., Pasik P., Pasik T. (1976). A Golgi study of neuronal types in the neostriatum of monkeys. Brain Res..

[21] Difiglia M., Pasik T., Pasik P. (1980). Ultrastructure of Golgi-impregnated and gold-toned spiny and aspiny neurons in the monkey neostriatum. J. Neurocytol..

[22] Cudkowicz M., Kowall N.W. (1990). Degeneration of pyramidal projection neurons in Huntington’s disease cortex. Ann. Neurol. Off. J. Am. Neurol. Assoc. Child Neurol. Soc..

[23] Hedreen J.C., Peyser C.E., Folstein S.E., Ross C.A. (1991). Neuronal loss in layers V and VI of cerebral cortex in Huntington’s disease. Neurosci. Lett..

[24] Sotrel A., Paskevich P., Kiely D., Bird E., Williams R., Myers R. (1991). Morphometric analysis of the prefrontal cortex in Huntington’s disease. Neurology.

[25] Levine M.S., Cepeda C., Hickey M.A., Fleming S.M., Chesselet M.-F. (2004). Genetic mouse models of Huntington’s and Parkinson’s diseases: Illuminating but imperfect. Trends Neurosci..

[26] Tobin A.J., Signer E.R. (2000). Huntington’s disease: The challenge for cell biologists. Trends Cell Biol..

[27] Menalled L.B. (2005). Knock-in mouse models of Huntington’s disease. NeuroRx.

[28] Reading S.A., Dziorny A.C., Peroutka L.A., Schreiber M., Gourley L.M., Yallapragada V., Rosenblatt A., Margolis R.L., Pekar J.J., Pearlson G.D. (2004). Functional brain changes in presymptomatic Huntington’s disease. Ann. Neurol. Off. J. Am. Neurol. Assoc. Child Neurol. Soc..

[29] Rosenblatt A., Abbott M.H., Gourley L.M., Troncoso J.C., Margolis R.L., Brandt J., Ross C.A. (2003). Predictors of neuropathological severity in 100 patients with Huntington’s disease. Ann. Neurol. Off. J. Am. Neurol. Assoc. Child Neurol. Soc..

[30] DiProspero N.A., Chen E.-Y., Charles V., Plomann M., Kordower J.H., Tagle D.A. (2004). Early changes in Huntington’s disease patient brains involve alterations in cytoskeletal and synaptic elements. J. Neurocytol..

[31] Li H., Li S.-H., Yu Z.-X., Shelbourne P., Li X.-J. (2001). Huntingtin aggregate-associated axonal degeneration is an early pathological event in Huntington’s disease mice. J. Neurosci..

[32] Reiner A., Albin R.L., Anderson K.D., D’Amato C.J., Penney J.B., Young A.B. (1988). Differential loss of striatal projection neurons in Huntington disease. Proc. Natl. Acad. Sci. USA.

[33] Weaver K.E., Richards T.L., Liang O., Laurino M.Y., Samii A., Aylward E.H. (2009). Longitudinal diffusion tensor imaging in Huntington’s Disease. Exp. Neurol..

[34] Rosas H.D., Lee S.Y., Bender A.C., Zaleta A.K., Vangel M., Yu P., Fischl B., Pappu V., Onorato C., Cha J.-H. (2010). Altered white matter microstructure in the corpus callosum in Huntington’s disease: Implications for cortical “disconnection”. Neuroimage.

[35] Klapstein G.J., Fisher R.S., Zanjani H., Cepeda C., Jokel E.S., Chesselet M.-F., Levine M.S. (2001). Electrophysiological and morphological changes in striatal spiny neurons in R6/2 Huntington’s disease transgenic mice. J. Neurophysiol..

[36] Bibb J.A., Yan Z., Svenningsson P., Snyder G.L., Pieribone V.A., Horiuchi A., Nairn A.C., Messer A., Greengard P. (2000). Severe deficiencies in dopamine signaling in presymptomatic Huntington’s disease mice. Proc. Natl. Acad. Sci. USA.

[37] Cepeda C., Ariano M.A., Calvert C.R., Flores-Hernández J., Chandler S.H., Leavitt B.R., Hayden M.R., Levine M.S. (2001). NMDA receptor function in mouse models of Huntington disease. J. Neurosci. Res..

[38] Laforet G.A., Sapp E., Chase K., McIntyre C., Boyce F.M., Campbell M., Cadigan B.A., Warzecki L., Tagle D.A., Reddy P.H. (2001). Changes in Cortical and Striatal Neurons Predict Behavioral and Electrophysiological Abnormalities in a Transgenic Murine Model of Huntington’s Disease. J. Neurosci..

[39] Ferrante R., Kowall N., Richardson E. (1991). Proliferative and degenerative changes in striatal spiny neurons in Huntington’s disease: A combined study using the section-Golgi method and calbindin D28k immunocytochemistry. J. Neurosci..

[40] Morfini G., Pigino G., Brady S.T. (2005). Polyglutamine expansion diseases: Failing to deliver. Trends Mol. Med..

[41] Wade A., Jacobs P., Morton A.J. (2008). Atrophy and degeneration in sciatic nerve of presymptomatic mice carrying the Huntington’s disease mutation. Brain Res..

[42] Jimenez-Sanchez M., Licitra F., Underwood B.R., Rubinsztein D.C. (2017). Huntington’s disease: Mechanisms of pathogenesis and therapeutic strategies. Cold Spring Harb. Perspect. Med..

[43] Kaemmerer W.F., Grondin R.C. (2019). The effects of huntingtin-lowering: What do we know so far?. Degener. Neurol. Neuromuscul. Dis..

[44] Dragatsis I., Dietrich P., Ren H., Deng Y., Del Mar N., Wang H., Johnson I., Jones K., Reiner A. (2018). Effect of early embryonic deletion of huntingtin from pyramidal neurons on the development and long-term survival of neurons in cerebral cortex and striatum. Neurobiol. Dis..

[45] Barnat M., Capizzi M., Aparicio E., Boluda S., Wennagel D., Kacher R., Kassem R., Lenoir S., Agasse F., Braz B.Y. (2020). Huntington’s disease alters human neurodevelopment. Science.

[46] Benn C.L., Sun T., Sadri-Vakili G., McFarland K.N., DiRocco D.P., Yohrling G.J., Clark T.W., Bouzou B., Cha J.-H.J. (2008). Huntingtin modulates transcription, occupies gene promoters in vivo, and binds directly to DNA in a polyglutamine-dependent manner. J. Neurosci..

[47] Biagioli M., Ferrari F., Mendenhall E.M., Zhang Y., Erdin S., Vijayvargia R., Vallabh S.M., Solomos N., Manavalan P., Ragavendran A. (2015). Htt CAG repeat expansion confers pleiotropic gains of mutant huntingtin function in chromatin regulation. Hum. Mol. Genet..

[48] Buckley N.J., Johnson R., Zuccato C., Bithell A., Cattaneo E. (2010). The role of REST in transcriptional and epigenetic dysregulation in Huntington’s disease. Neurobiol. Dis..

[49] Seong I.S., Woda J.M., Song J.-J., Lloret A., Abeyrathne P.D., Woo C.J., Gregory G., Lee J.-M., Wheeler V.C., Walz T. (2010). Huntingtin facilitates polycomb repressive complex 2. Hum. Mol. Genet..

[50] Zhai W., Jeong H., Cui L., Krainc D., Tjian R. (2005). In vitro analysis of huntingtin-mediated transcriptional repression reveals multiple transcription factor targets. Cell.

[51] Rogakou E.P., Sekeri-Pataryas K.E. (1999). Histone variants of H2A and H3 families are regulated during in vitro aging in the same manner as during differentiation. Exp. Gerontol..

[52] Banaszynski L.A., Allis C.D., Lewis P.W. (2010). Histone variants in metazoan development. Dev. Cell.

[53] Yadav T., Quivy J.-P., Almouzni G. (2018). Chromatin plasticity: A versatile landscape that underlies cell fate and identity. Science.

[54] Brownell J.E., Allis C.D. (1996). Special HATs for special occasions: Linking histone acetylation to chromatin assembly and gene activation. Curr. Opin. Genet. Dev..

[55] Lachner M., O’Sullivan R.J., Jenuwein T. (2003). An epigenetic road map for histone lysine methylation. J. Cell Sci..

[56] Jang H.S., Shin W.J., Lee J.E., Do J.T. (2017). CpG and non-CpG methylation in epigenetic gene regulation and brain function. Genes.

[57] Smith Z.D., Meissner A. (2013). DNA methylation: Roles in mammalian development. Nat. Rev. Genet..

[58] Maunakea A.K., Chepelev I., Cui K., Zhao K. (2013). Intragenic DNA methylation modulates alternative splicing by recruiting MeCP2 to promote exon recognition. Cell Res..

[59] Maunakea A.K., Nagarajan R.P., Bilenky M., Ballinger T.J., D’Souza C., Fouse S.D., Johnson B.E., Hong C., Nielsen C., Zhao Y. (2010). Conserved role of intragenic DNA methylation in regulating alternative promoters. Nature.

[60] Du Q., Luu P.-L., Stirzaker C., Clark S.J. (2015). Methyl-CpG-binding domain proteins: Readers of the epigenome. Epigenomics.

[61] Hudson N.O., Buck-Koehntop B.A. (2018). Zinc Finger Readers of Methylated DNA. Molecules.

[62] Zhu H., Wang G., Qian J. (2016). Transcription factors as readers and effectors of DNA methylation. Nat. Rev. Genet..

[63] Wu X., Inoue A., Suzuki T., Zhang Y. (2017). Simultaneous mapping of active DNA demethylation and sister chromatid exchange in single cells. Genes Dev..

[64] Wu X., Zhang Y. (2017). TET-mediated active DNA demethylation: Mechanism, function and beyond. Nat. Rev. Genet..

[65] Ito S., Shen L., Dai Q., Wu S.C., Collins L.B., Swenberg J.A., He C., Zhang Y. (2011). Tet proteins can convert 5-methylcytosine to 5-formylcytosine and 5-carboxylcytosine. Science.

[66] Kohli R.M., Zhang Y. (2013). TET enzymes, TDG and the dynamics of DNA demethylation. Nature.

[67] Kaas G.A., Zhong C., Eason D.E., Ross D.L., Vachhani R.V., Ming G.-L., King J.R., Song H., Sweatt J.D. (2013). TET1 controls CNS 5-methylcytosine hydroxylation, active DNA demethylation, gene transcription, and memory formation. Neuron.

[68] Li X., Wei W., Zhao Q.-Y., Widagdo J., Baker-Andresen D., Flavell C.R., D’Alessio A., Zhang Y., Bredy T.W. (2014). Neocortical Tet3-mediated accumulation of 5-hydroxymethylcytosine promotes rapid behavioral adaptation. Proc. Natl. Acad. Sci. USA.

[69] Hashimshony T., Zhang J., Keshet I., Bustin M., Cedar H. (2003). The role of DNA methylation in setting up chromatin structure during development. Nat. Genet..

[70] Lande-Diner L., Zhang J., Ben-Porath I., Amariglio N., Keshet I., Hecht M., Azuara V., Fisher A.G., Rechavi G., Cedar H. (2007). Role of DNA methylation in stable gene repression. J. Biol. Chem..

[71] Symmank J., Bayer C., Schmidt C., Hahn A., Pensold D., Zimmer-Bensch G. (2018). DNMT1 modulates interneuron morphology by regulating Pak6 expression through crosstalk with histone modifications. Epigenetics.

[72] Symmank J., Zimmer G. (2017). Regulation of neuronal survival by DNA methyltransferases. Neural Regen. Res..

[73] Marchese F.P., Raimondi I., Huarte M. (2017). The multidimensional mechanisms of long noncoding RNA function. Genome Biol..

[74] Zhao Y., Sun H., Wang H. (2016). Long noncoding RNAs in DNA methylation: New players stepping into the old game. Cell Biosci..

[75] Fan G., Beard C., Chen R.Z., Csankovszki G., Sun Y., Siniaia M., Biniszkiewicz D., Bates B., Lee P.P., Kühn R. (2001). DNA hypomethylation perturbs the function and survival of CNS neurons in postnatal animals. J. Neurosci..

[76] Rhee K., Yu J., Zhao C., Fan G., Yang X. (2012). Dnmt1-dependent DNA methylation is essential for photoreceptor terminal differentiation and retinal neuron survival. Cell Death Dis..

[77] Hutnick L.K., Golshani P., Namihira M., Xue Z., Matynia A., Yang X.W., Silva A.J., Schweizer F.E., Fan G. (2009). DNA hypomethylation restricted to the murine forebrain induces cortical degeneration and impairs postnatal neuronal maturation. Hum. Mol. Genet..

[78] Pensold D., Symmank J., Hahn A., Lingner T., Salinas-Riester G., Downie B.R., Ludewig F., Rotzsch A., Haag N., Andreas N. (2017). The DNA methyltransferase 1 (DNMT1) controls the shape and dynamics of migrating POA-derived interneurons fated for the murine cerebral cortex. Cereb. Cortex.

[79] Symmank J., Bayer C., Reichard J., Pensold D., Zimmer-Bensch G. (2020). Neuronal Lhx1 expression is regulated by DNMT1-dependent modulation of histone marks. Epigenetics.

[80] Symmank J., Gölling V., Gerstmann K., Zimmer G. (2019). The transcription factor LHX1 regulates the survival and directed migration of POA-derived cortical interneurons. Cereb. Cortex.

[81] Wei C.-W., Luo T., Zou S.-S., Wu A.-S. (2018). The Role of Long Noncoding RNAs in Central Nervous System and Neurodegenerative Diseases. Front. Behav. Neurosci..

[82] Lipton S.A., Kater S.B. (1989). Neurotransmitter regulation of neuronal outgrowth, plasticity and survival. Trends Neurosci..

[83] Santoro S.W., Dulac C. (2012). The activity-dependent histone variant H2BE modulates the life span of olfactory neurons. Elife.

[84] Guo J.U., Ma D.K., Mo H., Ball M.P., Jang M.-H., Bonaguidi M.A., Balazer J.A., Eaves H.L., Xie B., Ford E. (2011). Neuronal activity modifies the DNA methylation landscape in the adult brain. Nat. Neurosci..

[85] Wenderski W., Maze I. (2016). Histone turnover and chromatin accessibility: Critical mediators of neurological development, plasticity, and disease. BioEssays.

[86] Chen L.-F., Lin Y.T., Gallegos D.A., Hazlett M.F., Gómez-Schiavon M., Yang M.G., Kalmeta B., Zhou A.S., Holtzman L., Gersbach C.A. (2019). Enhancer histone acetylation modulates transcriptional bursting dynamics of neuronal activity-inducible genes. Cell Rep..

[87] Monahan K., Lomvardas S. (2012). Neuroscience: How keeping active pays off in the olfactory system. Elife.

[88] Pasantes-Morales H., Tuz K. (2006). Volume changes in neurons: Hyperexcitability and neuronal death. Mechanisms and Significance of Cell Volume Regulation.

[89] Levenson J.M., Roth T.L., Lubin F.D., Miller C.A., Huang I.-C., Desai P., Malone L.M., Sweatt J.D. (2006). Evidence that DNA (cytosine-5) methyltransferase regulates synaptic plasticity in the hippocampus. J. Biol. Chem..

[90] Nelson E.D., Kavalali E.T., Monteggia L.M. (2008). Activity-dependent suppression of miniature neurotransmission through the regulation of DNA methylation. J. Neurosci..

[91] Meadows J.P., Guzman-Karlsson M.C., Phillips S., Holleman C., Posey J.L., Day J.J., Hablitz J.J., Sweatt J.D. (2015). DNA methylation regulates neuronal glutamatergic synaptic scaling. Sci. Signal..

[92] Meadows J.P., Guzman-Karlsson M.C., Phillips S., Brown J.A., Strange S.K., Sweatt J.D., Hablitz J.J. (2016). Dynamic DNA methylation regulates neuronal intrinsic membrane excitability. Sci. Signal..

[93] Rudenko A., Dawlaty M.M., Seo J., Cheng A.W., Meng J., Le T., Faull K.F., Jaenisch R., Tsai L.-H. (2013). Tet1 is critical for neuronal activity-regulated gene expression and memory extinction. Neuron.

[94] Yu H., Su Y., Shin J., Zhong C., Guo J.U., Weng Y.-L., Gao F., Geschwind D.H., Coppola G., Ming G.-L. (2015). Tet3 regulates synaptic transmission and homeostatic plasticity via DNA oxidation and repair. Nat. Neurosci..

[95] Pensold D., Reichard J., Van Loo K.M.J., Ciganok N., Hahn A., Bayer C., Liebmann L., Gross J., Tittelmeier J., Lingner T. (2020). DNA Methylation-Mediated Modulation of Endocytosis as Potential Mechanism for Synaptic Function Regulation in Murine Inhibitory Cortical Interneurons. Cereb. Cortex.

[96] Schulz D.J., Baines R.A., Hempel C.M., Li L., Liss B., Misonou H. (2006). Cellular excitability and the regulation of functional neuronal identity: From gene expression to neuromodulation. J. Neurosci..

[97] Bayer C., Pitschelatow G., Hannemann N., Linde J., Reichard J., Pensold D., Zimmer-Bensch G. (2020). The DNA methyltransferase 1 (DNMT1) acts on neurodegeneration by modulating intracellular processes implicated in proteostasis. BioRxiv.

[98] Colacurcio D.J., Pensalfini A., Jiang Y., Nixon R.A. (2018). Dysfunction of autophagy and endosomal-lysosomal pathways: Roles in pathogenesis of Down syndrome and Alzheimer’s Disease. Free Radic. Biol. Med..

[99] Blanpied T.A., Scott D.B., Ehlers M.D. (2003). Age-related regulation of dendritic endocytosis associated with altered clathrin dynamics. Neurobiol. Aging.

[100] Alsaqati M., Thomas R.S., Kidd E.J. (2018). Proteins involved in endocytosis are upregulated by ageing in the normal human brain: Implications for the development of alzheimer’s disease. J. Gerontol. Ser. A.

[101] Stavoe A.K., Holzbaur E.L. (2020). Neuronal autophagy declines substantially with age and is rescued by overexpression of WIPI2. Autophagy.

[102] Cha J.-H.J. (2007). Transcriptional signatures in Huntington’s disease. Prog. Neurobiol..

[103] van Mierlo G., Veenstra G.J.C., Vermeulen M., Marks H. (2019). The complexity of PRC2 subcomplexes. Trends Cell Biol..

[104] von Schimmelmann M., Feinberg P.A., Sullivan J.M., Ku S.M., Badimon A., Duff M.K., Wang Z., Lachmann A., Dewell S., Ma’ayan A. (2016). Polycomb repressive complex 2 (PRC2) silences genes responsible for neurodegeneration. Nat. Neurosci..

[105] Sadri-Vakili G., Bouzou B., Benn C.L., Kim M.-O., Chawla P., Overland R.P., Glajch K.E., Xia E., Qiu Z., Hersch S.M. (2007). Histones associated with downregulated genes are hypo-acetylated in Huntington’s disease models. Hum. Mol. Genet..

[106] Kim M.-O., Chawla P., Overland R.P., Xia E., Sadri-Vakili G., Cha J.-H.J. (2008). Altered histone monoubiquitylation mediated by mutant huntingtin induces transcriptional dysregulation. J. Neurosci..

[107] Bannister A.J., Kouzarides T. (2011). Regulation of chromatin by histone modifications. Cell Res..

[108] Steffan J.S., Kazantsev A., Spasic-Boskovic O., Greenwald M., Zhu Y.Z., Gohler H., Wanker E.E., Bates G.P., Housman D.E., Thompson L.M. (2000). The Huntington’s disease protein interacts with p53 and CREB-binding protein and represses transcription. Proc. Natl. Acad. Sci. USA.

[109] Nucifora F.C., Sasaki M., Peters M.F., Huang H., Cooper J.K., Yamada M., Takahashi H., Tsuji S., Troncoso J., Dawson V.L. (2001). Interference by huntingtin and atrophin-1 with cbp-mediated transcription leading to cellular toxicity. Science.

[110] McFarland K.N., Huizenga M.N., Darnell S.B., Sangrey G.R., Berezovska O., Cha J.-H.J., Outeiro T.F., Sadri-Vakili G. (2014). MeCP2: A novel Huntingtin interactor. Hum. Mol. Genet..

[111] Zachariah R.M., Rastegar M. (2012). Linking epigenetics to human disease and Rett syndrome: The emerging novel and challenging concepts in MeCP2 research. Neural Plast..

[112] Chahrour M., Jung S.Y., Shaw C., Zhou X., Wong S.T., Qin J., Zoghbi H.Y. (2008). MeCP2, a key contributor to neurological disease, activates and represses transcription. Science.

[113] Fuks F., Hurd P.J., Wolf D., Nan X., Bird A.P., Kouzarides T. (2003). The methyl-CpG-binding protein MeCP2 links DNA methylation to histone methylation. J. Biol. Chem..

[114] Nan X., Ng H.-H., Johnson C.A., Laherty C.D., Turner B.M., Eisenman R.N., Bird A. (1998). Transcriptional repression by the methyl-CpG-binding protein MeCP2 involves a histone deacetylase complex. Nature.

[115] Ng C.W., Yildirim F., Yap Y.S., Dalin S., Matthews B.J., Velez P.J., Labadorf A., Housman D.E., Fraenkel E. (2013). Extensive changes in DNA methylation are associated with expression of mutant huntingtin. Proc. Natl. Acad. Sci. USA.

[116] Villar-Menéndez I., Blanch M., Tyebji S., Pereira-Veiga T., Albasanz J.L., Martín M., Ferrer I., Pérez-Navarro E., Barrachina M. (2013). Increased 5-methylcytosine and decreased 5-hydroxymethylcytosine levels are associated with reduced striatal A 2A R levels in Huntington’s disease. Neuromol. Med..

[117] Wood H. (2013). Altered DNA methylation and RNA splicing could be key mechanisms in Huntington disease. Nat. Rev. Neurol..

[118] Wang F., Yang Y., Lin X., Wang J.-Q., Wu Y.-S., Xie W., Wang D., Zhu S., Liao Y.-Q., Sun Q. (2013). Genome-wide loss of 5-hmC is a novel epigenetic feature of Huntington’s disease. Hum. Mol. Genet..

[119] Xu G.-L., Wong J. (2015). Oxidative DNA demethylation mediated by Tet enzymes. Natl. Sci. Rev..

[120] Rose N.R., Klose R.J. (2014). Understanding the relationship between DNA methylation and histone lysine methylation. Biochim. Biophys. Acta (Bba)-Gene Regul. Mech..

[121] Lehnertz B., Ueda Y., Derijck A.A., Braunschweig U., Perez-Burgos L., Kubicek S., Chen T., Li E., Jenuwein T., Peters A.H. (2003). Suv39h-mediated histone H3 lysine 9 methylation directs DNA methylation to major satellite repeats at pericentric heterochromatin. Curr. Biol..

[122] Bai G., Cheung I., Shulha H.P., Coelho J.E., Li P., Dong X., Jakovcevski M., Wang Y., Grigorenko A., Jiang Y. (2015). Epigenetic dysregulation of hairy and enhancer of split 4 (HES4) is associated with striatal degeneration in postmortem Huntington brains. Hum. Mol. Genet..

[123] Thomas E.A. (2016). DNA methylation in Huntington’s disease: Implications for transgenerational effects. Neurosci. Lett..

[124] Gutierrez A., Corey-Bloom J., Thomas E.A., Desplats P. (2020). Evaluation of Biochemical and Epigenetic Measures of Peripheral Brain-Derived Neurotrophic Factor (BDNF) as a Biomarker in Huntington’s Disease Patients. Front. Mol. Neurosci..

[125] Zuccato C., Ciammola A., Rigamonti D., Leavitt B.R., Goffredo D., Conti L., MacDonald M.E., Friedlander R.M., Silani V., Hayden M.R. (2001). Loss of huntingtin-mediated BDNF gene transcription in Huntington’s disease. Science.

[126] Pan Y., Daito T., Sasaki Y., Chung Y.H., Xing X., Pondugula S., Swamidass S.J., Wang T., Kim A.H., Yano H. (2016). Inhibition of DNA methyltransferases blocks mutant huntingtin-induced neurotoxicity. Sci. Rep..

[127] Francelle L., Lotz C., Outeiro T., Brouillet E., Merienne K. (2017). Contribution of neuroepigenetics to Huntington’s disease. Front. Hum. Neurosci..

[128] Valor L.M., Guiretti D. (2014). What’s wrong with epigenetics in Huntington’s disease?. Neuropharmacology.

[129] Valor L.M. (2015). Epigenetic-based therapies in the preclinical and clinical treatment of Huntington’s disease. Int. J. Biochem. Cell Biol..

